# Phylogeographical analysis shows the need to protect the wild yaks' last refuge in Nepal

**DOI:** 10.1002/ece3.7660

**Published:** 2021-05-14

**Authors:** Naresh Kusi, Prajwol Manandhar, Helen Senn, Jyoti Joshi, Muhammad Ghazali, Krishna Dev Hengaju, Sanej Prasad Suwal, Tshiring Lhamu Lama, Laxman Prasad Poudyal, Madhuri Thapa, Geraldine Werhahn

**Affiliations:** ^1^ Resources Himalaya Foundation Lalitpur Nepal; ^2^ Center for Molecular Dynamics Nepal Kathmandu Nepal; ^3^ WildGenes Laboratory Royal Zoological Society of Scotland Edinburgh UK; ^4^ IUCN Nepal Lalitpur Nepal; ^5^ Nature Conservation and Study Centre Kathmandu Nepal; ^6^ Snow Leopard Journeys Kathmandu Nepal; ^7^ Department of National Parks and Wildlife Conservation Kathmandu Nepal; ^8^ Department of Forests and Soil Conservation Kathmandu Nepal; ^9^ Wildlife Conservation Research Unit Department of Zoology University of Oxford Tubney UK

**Keywords:** *Bos mutus*, domestic yak, Humla, line transects, Nepal, threats, wild yak conservation

## Abstract

The wild yak *Bos mutus* was believed to be regionally extinct in Nepal for decades until our team documented two individuals from Upper Humla, north‐western Nepal, in 2014. The International Union for Conservation of Nature (IUCN) seeks further evidence for the conclusive confirmation of that sighting. We conducted line transects and opportunistic sign surveys in the potential wild yak habitats of Humla, Dolpa, and Mustang districts between 2015 and 2017 and collected genetic samples (present and historic) of wild and domestic yaks *Bos grunniens*. We also sighted another wild yak in Upper Humla in 2015. Phylogenetic and haplotype network analyses based on mitochondrial D‐loop sequences (~450 bp) revealed that wild yaks in Humla share the haplotype with wild yaks from the north‐western region of the Qinghai‐Tibetan Plateau in China. While hybridization with domestic yaks is a major long‐term threat, illegal hunting for meat and trophy put the very small populations of wild yaks in Nepal at risk. Our study indicates that the unprotected habitat of Upper Humla is the last refuge for wild yaks in Nepal. We recommend wild yak conservation efforts in the country to focus on Upper Humla by (i) assigning a formal status of protected area to the region, (ii) raising awareness in the local communities for wild yak conservation, and (iii) providing support for adaptation of herding practice and pastureland use to ensure the viability of the population.

## Graphical abstract

Our study carries a global relevance as it presents phylogenetic and haplotype network analyses of wild and domestic yak samples to reveal that the wild yaks in Humla, Nepal, share the haplotype with wild yaks from the north‐western region of the Qinghai‐Tibetan Plateau in China, providing validation to the wild yak rediscovery in Nepal that was based on morphology and behavior of the sighted animals. We also derive phylogeographical inference for the wild yaks in the Nepalese Transhimalayas and discuss their hybridization status while also outlining the conservation implications for the wild yaks that are critically endangered in the country.

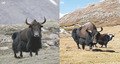

## 1 INTRODUCTION

The wild yak *Bos mutus* is globally listed as Vulnerable (VU) by the IUCN Red list (Buzzard & Berger, 2016). This ungulate inhabits the alpine tundra, grasslands, and cold desert regions of the north‐western Qinghai‐Tibetan Plateau (QTP) and adjacent high‐altitude regions (Wiener et al., 2003) at elevations between 3,000 and 6,100 masl (Han, 2014; Leslie & Schaller, 2009). Its geographical distribution once ranged from the northern Transhimalayan habitats in Nepal, Bhutan, and India in the Hindu Kush Himalaya and across the QTP in China (Zhao & Gao, 1991 cited in Han, 2014). Schaller & Liu (1996) updated the distribution to include the Tibetan Plateau habitats in China, India, and Nepal, whereas (Harris & Leslie, 2008) considered the species to be present only in China and India, while regionally extinct in Bhutan and Nepal. At present, around 10,000–15,000 wild yaks roam QTP in an area of approximately 400,000 km^2^ (Buzzard & Berger, 2016; Schaller, 1998; Zhang et al., 2020).

For many decades, only indirect evidence like horns, skulls, and pelts of presumed wild yaks indicated their past and/or current presence in Nepal. The Transhimalayan wild yak habitats in Nepal are contiguous with the Tibetan Plateau of the Tibetan Autonomous Region (TAR) in China as they represent parts of the western end of the Plateau. This allows seasonal movement of wild yaks from TAR into northern Nepal (Miller et al., 1994). However, due to the lack of evidence of live animals, Jnawali et al. (2011) assessed the species as “data deficient” and “possibly regionally extinct” in Nepal. Wild yaks are a protected priority species in the country (GoN, 1973) and are listed in Appendix I of the Convention on International Trade in Endangered Species of Wild Fauna and Flora (CITES) (Buzzard & Berger, 2016).

The retaliatory killing of wild yaks (usually bulls) by livestock herders to prevent hybrid offspring and abductions of female domestic yaks *Bos grunniens* seems the most evident threat to wild yaks in Nepal (F. Tamang and G. Lama pers comm 2015). The herders and their domestic livestock threaten the wild yaks further through habitat encroachment and displacement (Harris, 2007), while the possibility of disease transmission between wild–domestic yak interfaces remains as a substantial threat (Buzzard & Berger, 2016; Schaller & Liu, 1996). Illegal hunting for meat and trophy adds to the threats (F. Tamang and G. Lama pers comm 2015).

In 2014, we sighted two wild yaks in the remote Transhimalayan valleys of Upper Humla in north‐western Nepal, leading to the rediscovery of the species in the country (Acharya et al., 2015). National and international experts identified the sighted wild yaks by referring to their morphology and behavior from our photographs and video footage. Some experts also suggested to perform genetic analyses for conclusive identification. As such, IUCN considers our sighting record as uncertain and seeks for an additional evidence for conclusive confirmation (Buzzard & Berger, 2016).

The mitochondrial D‐loop or control region has been widely used for investigating intraspecific genetic variation, population structure, and demographic histories of animal domestication (Beja‐pereira et al., 2004; Guo et al., 2006; Jansen et al., 2002; Lai et al., 2007; Larson et al., 2005; Luikart et al., 2001; Troy et al., 2001; Wolf, 1999). This is because D‐loop is the most variable region of mtDNA having high nucleotide polymorphism compared to the rest of the regions in mtDNA. For example, the variable sites in the D‐loop of yak mtDNA are seven times more than that observed in the entire yak mtDNA (Wang et al., 2010). The genetic diversity of domestic and wild yaks in the QTP has been inferred through phylogeographical studies based on the D‐loop region (Guo et al., 2006; Wang et al., 2010; Ma et al., 2010). Wang et al. (2010) derived distinct phylogeographical differences between wild and domestic yaks based on the D‐loop sequence data. We chose the mitochondrial D‐loop data to discern the genetic identity of our wild yak sightings by comparing our samples with the reference data from the previous studies. However, mtDNA being maternally inherited is a haploid and nonrecombining locus and thus cannot present evidence of admixture or hybridization. But the presence of reference dataset (about wild versus domestic haplotypes) allows us to utilize the mitochondrial D‐loop data to study phylogeographic patterns and domestication histories.

## 2 MATERIALS AND METHODS

### 2.1 Study area

We conducted field researches in Upper Humla, Upper Dolpa, and Upper Mustang, all located in the Transhimalayan belt of north‐western Nepal, during the spring and summer seasons of 2015–2017. Upper Humla is currently outside the protected area system, Upper Dolpa constitutes large part of the Shey‐Phoksundo National Park (SPNP), and Upper Mustang lies entirely within the Annapurna Conservation Area (ACA). All the three study areas share an international border with the TAR of China. Elevation in the study areas ranged between 3,600 and 5,600 masl, and the vegetation is characterized by alpine grasslands and steppes interspersed with patches of shrubland (Miehe et al., 2016). Local communities, belonging to the Tibetan ethnic group, are mostly agro‐pastoralists who graze domestic yaks, cattle *Bos taurus, dzos/*jhoppas (yak‐cattle hybrids) *Bos* spp., horses *Equus ferus coballus,* goats *Capra* *aegagrus hircus*, and sheep *Ovies aries* in the alpine pastures during spring and summer seasons. They shift the livestock herds among pasturelands and bring the animals down to the villages during winter (Bauer, 2004). According to the latest national population and housing census of Nepal (2011), there were a total of 2,758 domestic yaks in Humla (area: 5,655 km^2^), 7,517 in Dolpa (area: 7,889 km^2^), and 2008 in Mustang District (area: 3,573 km^2^) (MOLD, 2017).

### 2.2 Noninvasive genetic sampling

**FIGURE 1:**
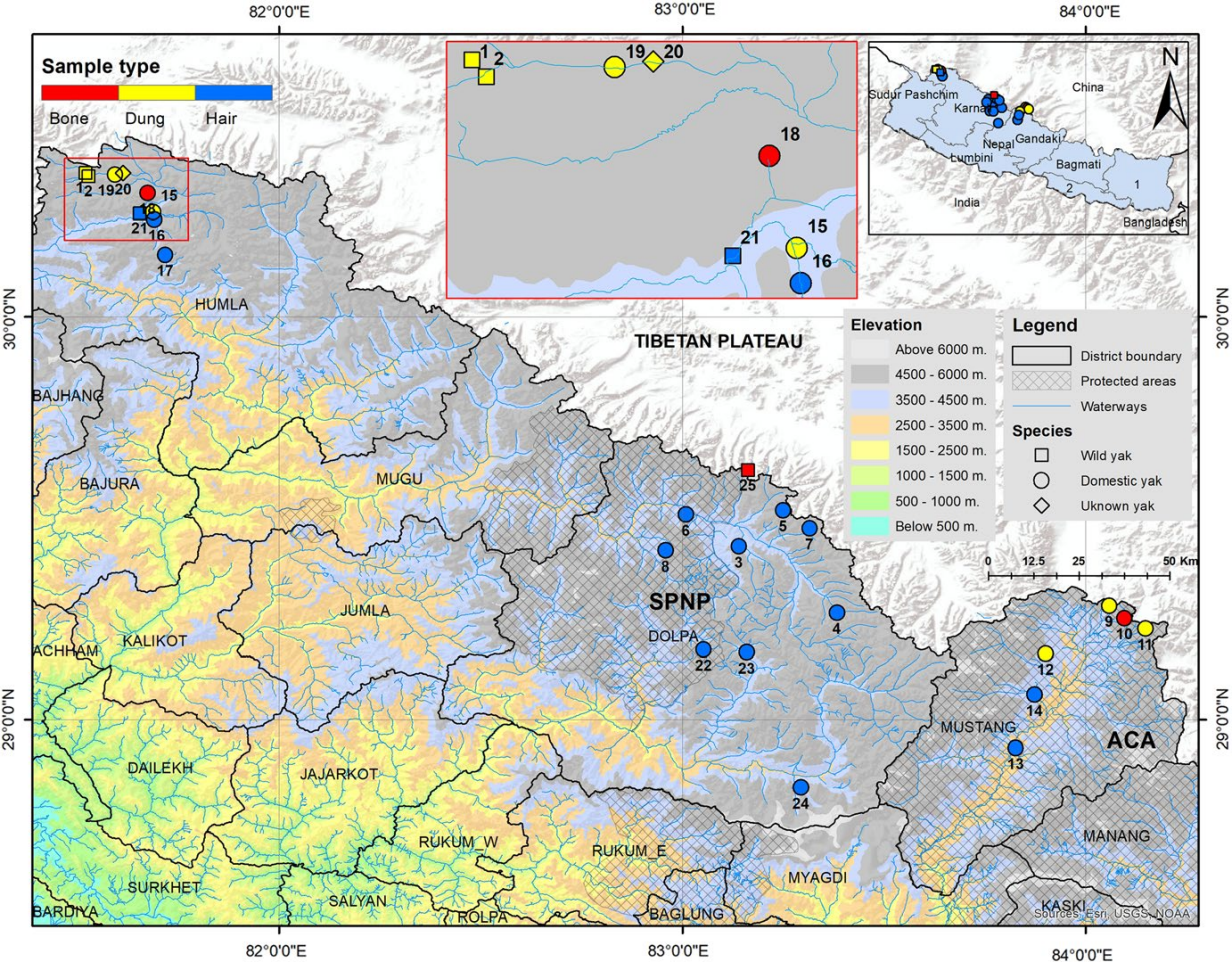
Locations of yak samples collected in this study. Mustang lies within the Annapurna Conservation Area (ACA) and Dolpa within the Shey‐Phoksundo National Park (SPNP); Humla lies outside the protected area system of Nepal. Numbers 1–25 are the serial numbers (SN) of the samples as they appear in Table S1. A zoomed‐in view of the samples collected in Upper Humla is provided in the inset

We conducted line transects (Nichols & Karanth, 2005) and opportunistic sign surveys in the potential areas to look for any historical or present evidence of wild yaks. We noted geographical locations of all places (e.g., monasteries, local houses, mountain passes, and other natural features), with a GPS device (GPSMAP 64s), where we retrieved evidence (e.g., body parts such as horn, skull, pelt) of presumed wild yaks (Figure 1). We collected genetic samples from these wild yak specimens (bone from horn and skull and hair with hair bulb from pelt) and noninvasive dung samples from live wild yaks. We also collected dung, hair, and bone samples from domestic yaks. We stored the dung samples in 2‐ml cryo‐vials containing DET buffer and the bone and hair samples in 15‐ml sampling vials containing silica gels with a small layer of cotton atop the silica, before transferring them to the Intrepid Nepal Lab in Kathmandu, Nepal, where we performed the genetic analyses.

We collected 28 samples: six of presumed wild yak (three bone samples, two dung samples, and one hair sample), 21 of presumed domestic yak: (five dung samples, 14 hair samples, and two bone samples), and one unknown dung sample. We obtained genetic data from 25 samples, while two bone samples of suspected wild yaks and one hair sample of domestic yak failed possibly due to poor sample quality (see Table S1 for details). We treated the samples to be from wild yaks based on their morphology, that is, long hair and large skeleton (Wang et al., 2010), and the local history, that is, reports stating their origin from wild yaks.

### 2.3 Genetic analysis

We extracted DNA off the genetic samples and generated partial D‐loop (~450 bp) sequences of the mitochondrial genome using self‐designed primers (YAK_Dloop2_F: 5′‐ GAGCCTCACCAGTATTAAATTT‐3′ and YAK_Dloop2_R: 5′‐ACAGTTATGTGTGAGCATGGGC‐3′). Details on the laboratory methods are provided in the supplementary information. We retrieved a subset of reference sequences of wild (*n* = 34) and domestic (*n* = 96) yaks from QTP from NCBI GenBank (See Table S2). This dataset represented all D‐loop haplogroups of wild and domestic yaks generated by Wang et al. (2010) who inferred phylogeographical structure of the yaks across their range in QTP. We then compared the 25 sequences generated in this study (Table S1) with the reference sequence dataset by aligning them using MUSCLE (Edgar, 2004) and visually inspected the alignment in AliView (Larsson, 2014). We drew haplotype networks among the D‐loop haplotype sequences from Nepal and China with PopART (Leigh & Bryant, 2015) using median‐joining networks (Bandelt et al., 1999). We performed a phylogenetic analysis of all yak D‐loop haplotype sequences with the Bayesian inference (BI) method using Markov Chain Monte Carlo (MCMC) in MrBayes v3.2.2 (Ronquist et al., 2012). We included *Bison* *bison* sequence (GenBank accessions: U12936) as an outgroup to root all yak D‐loop haplotypes. We performed two parallel runs of four chains (three heated and one cold) for 3,000,000 generations, with sampling done at every 500th generation. We diagnosed the convergence of the Bayesian posterior probabilities of four chains in Tracer v1.7.1 (Rambaut et al., 2018) and visualized a summarized consensus tree using FigTree v1.4.2 (Rambaut, 2014).

We also collected informal information on historical and current presence of wild yaks and traditional use of their body parts (see Acharya et al. (2015) for details).

## 3 RESULTS

**FIGURE 2:**
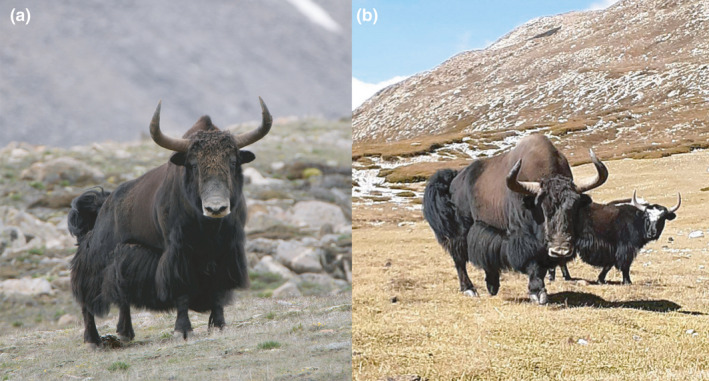
Photographs of wild yaks from Upper Humla. (a) The single wild yak seen in Upper Humla in July 2015. Its greyish‐white muzzle, handle‐bar horns, hump raised above the shoulder, long shaggy black‐dark brown coat and thick tail are characteristic of wild yaks. Note the dung pile between its hind legs. Photo: Naresh Kusi. (b) A wild yak sighted with a domestic yak in Upper Humla in September 2020. The domestic yak lacks a greyish‐white muzzle, has a nonuniform coat (with white patches on its forehead and shoulder), thinner and smaller horns, is smaller in size, and carries an inconspicuous hump. Photo: Bishnu Bahadur Lama

We encountered two live wild yak individuals in 2014 and a single wild yak in 2015 in Upper Humla (Figure 2a). But we did not find any evidence of live wild yaks in Upper Dolpa and Upper Mustang.

**FIGURE 3:**
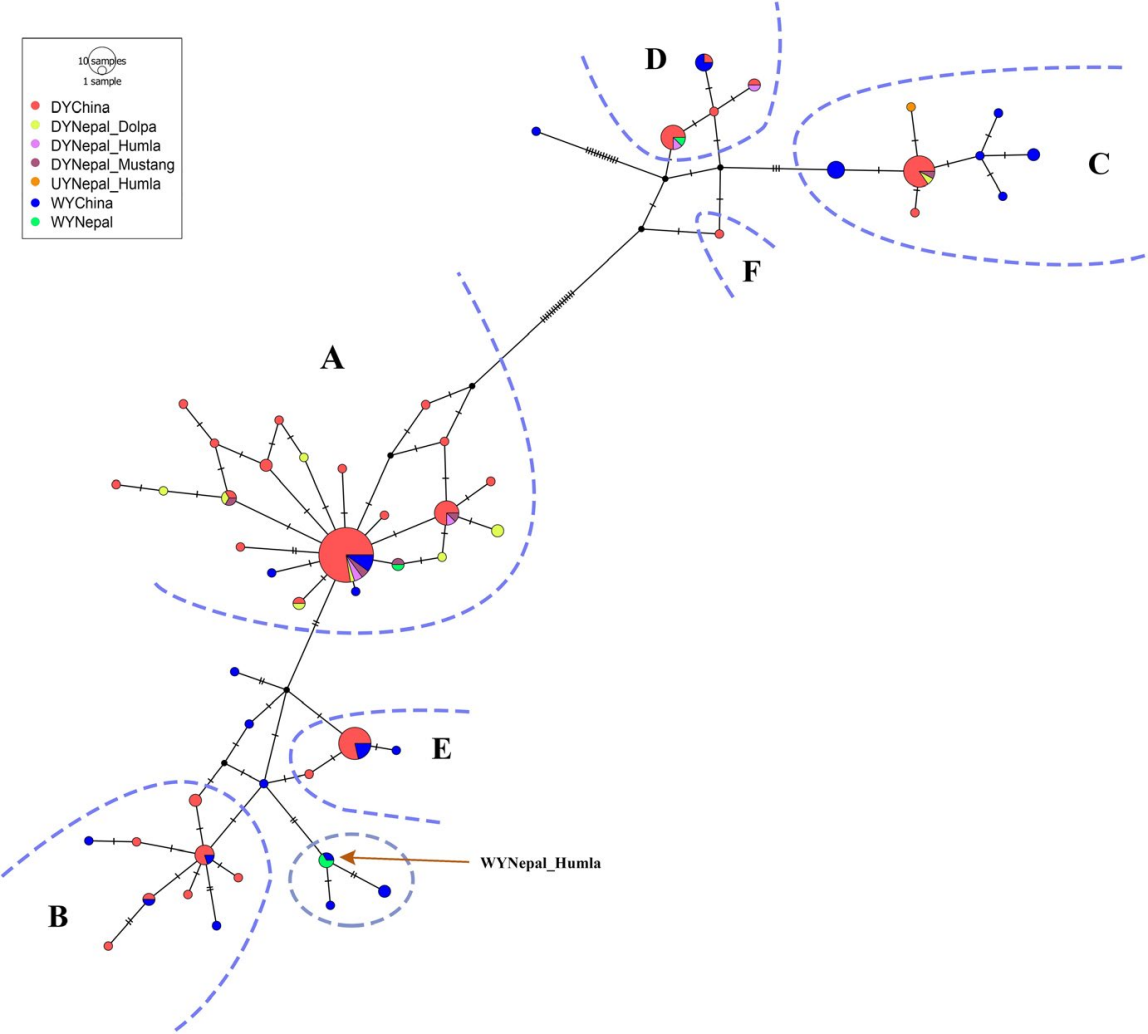
Haplotype network of D‐loop sequences of wild and domestic yaks in north‐western Nepal (this study) and western China (Wang et al., 2010). Each circle represents a haplotype while the area of the circle is proportional to its frequency. Different colors are used to indicate samples from different geographic regions. DY, WY, and UY indicate domestic yak, wild yak, and unknown yak, respectively. (a, b, c, d, e, f) represent different haplogroups as defined by previous studies (Guo et al., 2006; Wang et al., 2010). The haplotype “WYNepal_Humla” indicated by an arrow belongs to the wild yak sighted in Humla; this haplotype was previously identified in wild yak from north‐western QTP in China. The haplotype is directly associated with a small endemic wild haplogroup marked with a circular polygon

**FIGURE 4:**
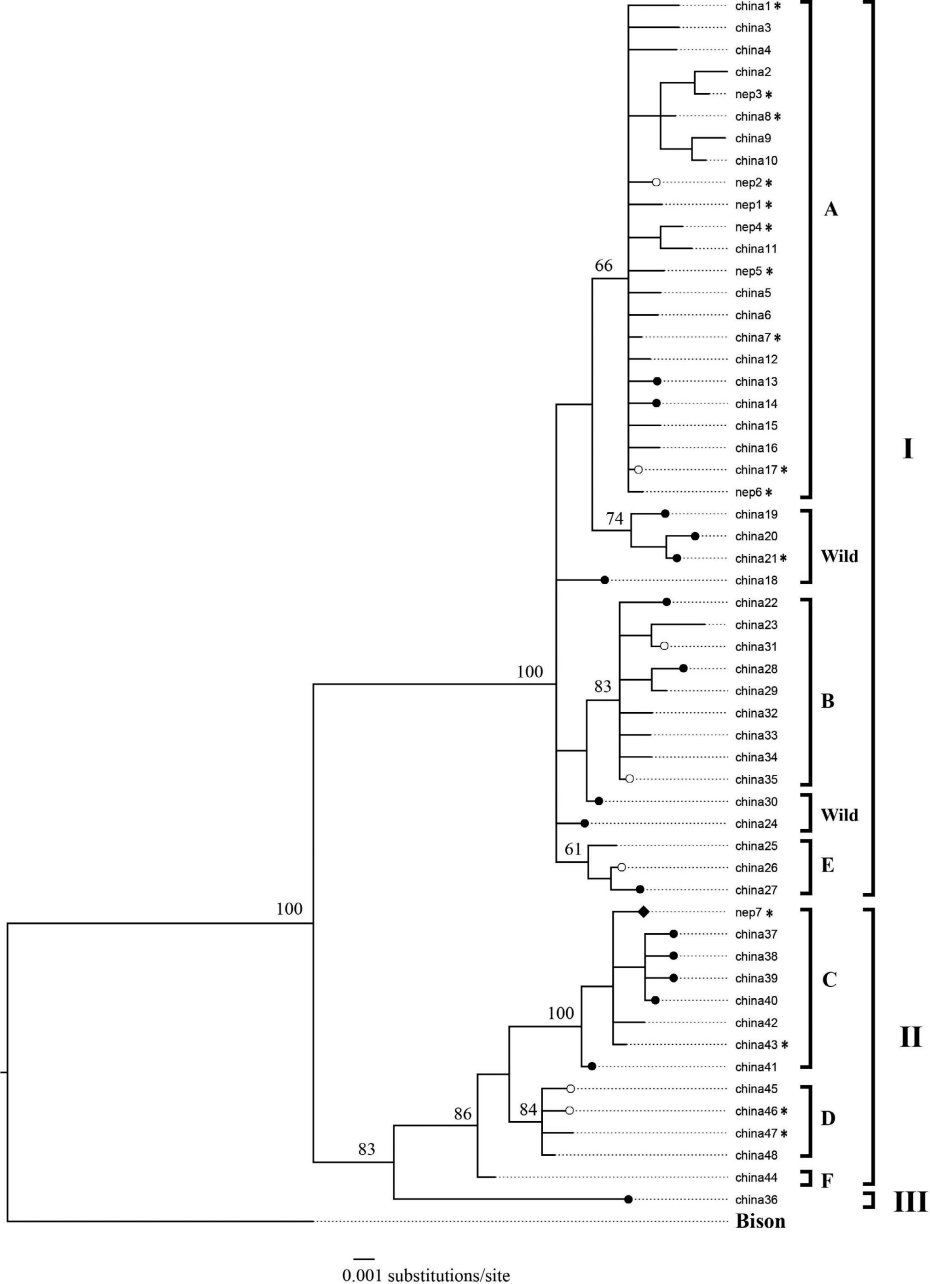
Phylogenetic tree of all domestic and wild yak D‐loop haplotypes/haplogroups in western China and north‐western Nepal (this study) constructed by Bayesian inference, rooted with Bison bison. The length of the alignment is 637 bp. The haplotypes from reference sequences are referred with prefix “china” while new haplotypes identified in this study are referred with prefix “nep.” Branches with filled circles indicate haplotypes found only in wild yaks; filled diamond indicates haplotype found in unknown yak; open circles are haplotypes shared by domestic and wild yaks; branches without circles are haplotypes identified only in domestic yaks. The haplotypes representing 25 yak samples from this study are marked with an asterisk. The wild yak sighted in Humla belonged to haplotype “china21” which is a wild yak haplotype previously identified in north‐western QTP in China. Support values at the nodes represent Bayesian posterior probabilities. Accession numbers are listed in Tables S1 and S2

For the final analyses, we used data from 25 yak samples (domestic yaks: 20, wild yaks: 4, and unknown yak: (1) in Nepal and 130 yak sequences (domestic yaks: 96, wild yaks: 34) in China (Wang et al., 2010). We identified a total of 55 haplotypes from these 155 sequences, consisting of 48 haplotypes from published sequences of China and seven haplotypes newly identified from sequences of Nepal (Tables S1 and S2). Of the 25 yak sequences from Nepal, 16 sequences belonged to eight previously known haplotypes and nine sequences belonged to seven new haplotypes (Figures 3 and 4, and Table S2). The sequences generated in this study have been deposited in NCBI GenBank under accessions MW048416 to MW048440.

The haplotype network showed that the domestic yak haplotypes of Nepal are mostly distributed among three major haplogroups (A, C, and D) which are shared by domestic and wild yaks with the majority representing domestic yaks in terms of frequency (Figure 3). The topology of the phylogenetic tree distinguished three lineages (I, II, and III) of the yaks, with domestic yak haplogroups belonging in lineages I and II (Figure 4). Haplotypes of two historically presumed wild yak samples also belonged within haplogroup A (pelt kept in a house in Upper Humla) and haplogroup D (skull in a mountain pass in Upper Dolpa). The two dung piles of wild yak collected from two different locations (30.35707°N, 081.51979°E and 30.34817°N, 081.52723°E) within Upper Humla (samples with SN 1 and 2 in Figure 1) belonged to the same haplotype that was identical with a haplotype of wild yaks in north‐western QTP. This haplotype associated with an endemic haplogroup of wild yak within lineage I (circled in Figure 3; haplotype “china21” in Figure 4). The haplotype belonging to the dung sample of the unknown yak in Humla belonged within haplogroup C.

## 4 DISCUSSION

### 4.1 Phylogeographical structure and hybridization state

Wild yaks evolved into three allopatric lineages in different regions during the Pleistocene glaciation events (approximately 800,000–600,000 years ago) and reunited into a single gene pool during a postglacial population expansion and subsequent migrations before multiple domestications began across QTP in the Holocene (approximately 11,000 years ago) (Guo et al., 2006; Ho et al., 2008; Wang et al., 2010). Due to the recent and multiple domestication histories across the geographical range, haplotypes belonging to various morphological breeds of domestic yaks do not correlate to their contemporary geographical distribution across QTP. Multiple domestication events that occurred randomly throughout QTP beginning approximately 10,000 years ago resulted in majority of the haplogroups among the two lineages of yaks to consist of both domestic and wild haplotypes (Wang et al., 2010). The majority of yak haplotype diversity is dominated within the domestic yaks having a population of more than 14 million found across QTP, while wild yaks with estimated population size of around 10,000–15,000 animals are mostly confined to the north‐western region of QTP with some sparse distributions in southern and western regions of the Tibetan Plateau (Buzzard & Berger, 2016; Wiener et al., 2003; Zhang et al., 2020). Although it is difficult to distinguish between domestic and wild yaks based on mtDNA alone, some D‐loop haplotypes (such as in haplogroup C and the one consisting of “WYNepal_Humla”) distinctively represent wild yak gene pools.

Wang et al. (2010) revealed clear phylogeographical distinctions among the domestic and wild yak D‐loop haplotypes. Similarly, Wang et al. (2014) found a clearer distinction between domestic and wild yaks based on Single Nucleotide Polymorphisms (SNPs) genotyping across whole genome data of three domestic and three wild yaks. However, due to limited sample size and contrastingly different sampling regions of domestic and wild yaks in their study, it is still impossible to fully disentangle the distinctions they report.

A comprehensive approach for differentiating wild yaks from domestic yaks would be to combine evidences from morphological identifications and genetic analyses. The D‐loop haplotype generated from dung samples of wild yaks (collected from Upper Humla in 2015) were found to be identical to a haplotype belonging to an endemic haplogroup of wild yaks sampled in north‐western QTP by Wang et al. (2010). This particular haplotype is only five nucleotide mutations away from the next domestic animal, which is less than the variation observed among domestic haplotypes in haplogroups A and B (Figure 3). However, there are also many haplotypes that are unique to the wild yak gene pool which are at a relatively small genetic distance from the closest domestic haplotype. A broader and more comprehensive sampling and analysis involving a genome‐wide SNP‐based assessment will probably provide clearer insights into the distinction between wild and domestic yaks. Interestingly the location where the haplotype “WYNepal_Humla” is found lies approximately 1,000 km south‐west of north‐western QTP where the “China21” wild haplotype was identified. Most of this region (between Upper Humla (Nepal) and north‐western QTP) represent wilderness areas with no human settlements. So it is very unlikely that domestic yak herds can get transported between these points. This further suggests that “WYNepal_Humla” is a wild haplotype. Our data strongly suggests that the wild yak haplotype identified from Humla in this study is unique and rare among wild yaks and has a potential to get incorporated into domestic gene pool from hybridization. National and international experts agreed that the morphological features (greyish‐white muzzle, long, and shaggy black‐dark brown coat, thick tail, hump raised above the shoulder and handle‐bar horns) and behavior (they were very shy and ran away as soon as they saw humans) of the animals we sighted in 2014 are typical of wild yaks (see Acharya et al., 2015). A proper management intervention is urgent to formally protect the wild yak and its habitat in north‐western Nepal.

The other two haplotypes of presumed (historical) wild yaks collected from Humla (hair from pelt, sample age: approx. 5 years) and Dolpa (bone from skull, sample age: approx. 10 years) are identical to contemporary haplotypes belonging to domestic yaks in haplogroups A and D. Both of these haplogroups consist of haplotypes shared by both wild and domestic yaks, the majority of which belong to the domestic yak gene pool. Similarly, the haplotype of the unknown yak from Humla (dung) is associated within the haplogroup C which consist of haplotypes shared by both wild and domestic yaks with the majority belonging to wild yak gene pool. However, the haplotype shows a more direct affinity with domestic haplotypes within the haplogroup suggesting that it is the most likely to be of a domestic origin. These inferences clearly show the extent of hybridization across the wild yak range since the historic past. The other haplotypes of domestic yaks from Mustang, Dolpa and Humla are unevenly distributed among haplogroups A, C, and D irrespective of their geographic locations. These results corroborate the previous findings of Guo et al. (2006) and Wang et al. (2010) about the phylogeography of yaks and state of hybridizations between wild and domestic animals across their contemporary range.

Local yak herders in Upper Humla graze domestic yaks frequently in wild yak habitats. During the herding seasons, older wild yak bulls are known to visit the herder's camp to mate with domestic female yaks (Figure 2b). This lack of reproductive barrier between wild and domestic yaks allows them to hybridize. Similar trends have been observed in Asian wild water buffaloes *Bubalus arnee* whose wild population face serious threats due to hybridization with domestic water buffaloes *Bubalus bubalis* (Flamand et al., 2003; Scherf, 2000). Domestic yak bulls that mate with wild females can mediate genetic introgression into wild yak populations. From the perspective of genetic diversity, hybridization is potentially detrimental to wild yaks because frequent introgression will gradually change their genetic integrity and cause the wild gene pool to relinquish among the admixed ancestry in the longer term. However, the wild yak gene pool in Upper Humla, which consists of only a few individuals, is unlikely to get exposed to a severe introgression because the hybrid progeny (produced through mating of a wild male and domestic female) usually remains within the domestic herd that is a part of a bigger domestic population comprising thousands of animals. Importantly, herders do not prefer hybrid offspring as they are generally shyer and more difficult to handle (Buzzard & Berger, 2016). Extent of such hybridization footprints are best studied by using markers from mitochondrial as well as nuclear genomes. However, we have utilized mitochondrial marker (that are maternally inherited) only in this study by referring to Guo et al. (2006) and Wang et al. (2010). Moreover, the wild bulls are known to abduct domestic females at times (F. Tamang and G. Lama pers comm. 2015). The chances of higher genetic introgression from the domestic yaks into the wild yak gene pool may become evident only if the abducted domestic females remain permanently in the wild herds. But such incidents are rare because the herders will retrieve the abducted female domestic yaks in most cases. Nevertheless, the fact that our two presumed historic wild yak samples from Humla and Dolpa show haplotypes belonging to the haplogroups that are shared by both domestic and wild yaks (as observed in their range throughout QTP) deserves attention of conservation biologists because they could have been misidentified as wild yaks by the local people. They can be either feral domestic yaks or admixed yaks (showing phenotypic resemblance with wild yaks). Also, the endemic and unique haplotypes/haplogroups of wild yak are among the most vulnerable groups under direct threat from such hybridization events, meaning that future generations of the few individuals we sighted in Humla (that also belonged to these endemic wild haplogroups) are susceptible to hybridization.

### 4.2 Importance of habitat protection for wild yak conservation

Almost 10 wild yaks were killed in Upper Humla during the last decade (F. Tamang and G. Lama pers comm 2015). Anecdotal reports like these motivated deeper investigations into the matter and the present research. During our observations of wild yaks in 2014 and 2015, the animals were extremely shy of humans. This behavior may reflect the intense hunting pressure they face from humans. The large amount of meat resulting from a hunted wild yak and the high price of wild yak heads (approximately 1,000 USD) in the nearby illegal markets of TAR (Tibetans have a tradition of hanging a wild yak head by the entrance of their houses as a status symbol) act as incentives for killing the animals. The construction of motor roads in the Tibetan Plateau has further intensified the poaching risk (Shi et al., 2016) while also degrading the natural habitat of wild yaks. However, to date, the road network in Upper Humla spares the secluded valleys inhabited by wild yaks and it is of great importance to ensure that any future developmental activities spare these valleys that present valuable habitat refuges, especially for the wild yak.

Both in China and in India, wild yaks are confined to the boundaries of nature reserves (Shi et al., 2016), indicating the importance of protected areas in their conservation. Our findings suggest that Upper Humla is currently the last refuge to wild yaks in Nepal. The last remaining wild yaks of the country are under serious risk of going truly extinct if immediate steps are not taken to formally protect the area. Building on the recent reassessment of wild yaks in Nepal as “Critically Endangered” (Amin et al., 2018), a formal protection of this area will prevent the small population of wild yaks from facing further decline. This might also allow for recolonization of the area by more wild yaks from TAR leading to the formation of a small intact transboundary population in Nepal. Wild yak conservation efforts in the Nepalese Transhimalayas should incorporate sufficient and effective programs on raising awareness in the local communities and supporting them with measures to ensure habitat availability and population viability of the wild yaks. Simultaneous efforts are required to ensure the continuation of traditional agro‐pastoralist livelihood while conferring protection to wild yaks. Government‐level initiatives like creating habitat refuges for wild yaks while encouraging rotational grazing of domestic yaks in other available pastures, maintaining sustainable livestock numbers, and halting wild yak poaching and trade will support wild yak conservation in Nepal.

## CONFLICT OF INTEREST

The authors have no conflict of interest.

## AUTHOR CONTRIBUTIONS

**Naresh Kusi:** Conceptualization (lead); data curation (lead); formal analysis (equal); methodology (equal); writing‐original draft (lead); writing‐review & editing (equal). **Prajwol Manandhar:** Conceptualization (equal); data curation (equal); formal analysis (lead); methodology (equal); writing‐original draft (equal); writing‐review & editing (equal). **Helen** **Senn:** Methodology (equal); writing‐review & editing (equal). **Jyoti Joshi:** Investigation (equal); writing‐review & editing (equal). **Muhammad** **Ghazali:** Methodology (equal); writing‐review & editing (equal). **Krishna Dev** **Hengaju:** Investigation (equal); writing‐review & editing (equal). **Sanej Prasad Suwal:** Investigation (equal); writing‐review & editing (equal). **Tshiring Lhamu Lama:** Investigation (equal); writing‐review & editing (equal). **Laxman Prasad Poudyal:** Resources (equal); writing‐review & editing (equal). **Madhuri Thapa:** Resources (equal); writing‐review & editing (equal). **Geraldine** **Werhahn:** Conceptualization (equal); methodology (equal); writing‐original draft (equal); writing‐review & editing (equal).

## Supporting information

Supplementary Material

## Data Availability

All relevant data for this study are included in and accessible through this manuscript.
