# Supplementary material for: Phylogeographical analysis shows the need to protect the wild yaks' last refuge in Nepal
Source: Ecol Evol. 2021 May 14;11(12):8310–8. doi: 10.1002/ece3.7660 (PMC8216926; doi:10.1002/ece3.7660)
Supplement: Supplementary file 1 — Supplementary Material [file ECE3-11-8310-s001.docx]

**Supplementary Information for:**

**Phylogeographical analysis shows the need to protect the wild yaks’ last refuge in Nepal**

Naresh Kusi^1*^, Prajwol Manandhar^2*^, Helen Senn^3^, Jyoti Joshi^2^ , Muhammad Ghazali^3^, Krishna Dev Hengaju^4^, Sanej Prasad Suwal^5^, Tshiring Lhamu Lama^6^, Laxman Prasad Poudyal^7^, Madhuri Thapa^8^ and Geraldine Werhahn^9^

^1^ Resources Himalaya Foundation, Sanepa, Lalitpur, Nepal

^2^ Center for Molecular Dynamics Nepal, Kathmandu, Nepal

^3^ WildGenes Laboratory, Royal Zoological Society of Scotland, Edinburgh, EH12 6TS, UK

^4^ IUCN Nepal, Kupondol, Lalitpur, Nepal

^5^ Nature Conservation and Study Center, Kathmandu, Nepal

^6^ Snow Leopard Journeys, Kathmandu, Nepal

^7^ Department of National Parks and Wildlife Conservation, Kathmandu, Nepal

^8^ Department of Forests and Soil Conservation, Kathmandu, Nepal

^9^ Wildlife Conservation Research Unit, Department of Zoology, University of Oxford, Tubney House, Abingdon Road, Tubney, OX13 5QL, UK

**Lab methods**

**DNA Extraction**

**Dung samples:**

DNA from dung (swab in DET buffer) was extracted using QIAamp Fast DNA Mini Stool Kit (Qiagen, Germany). The swab was turned around (swab end up) and centrifuged for 1 min at 5,000 RPM. The supernatant was pipetted in a clean 1.5 ml Ep tube (~200 µl). 1000 µl of inhibitEX solution was added and vortexed for one minute and left at room temperature for a minute to allow the inhibitors to be absorbed. The tube was centrifuged for one minute at full speed (13.5 * 1000 RPM) to pellet the remaining fecal particles. 600 µl supernatant was collected in fresh 2 ml tube and 25 µl Proteinase K was added to the centrifuged supernatant (600 µl) to which another 600µL of Lysis buffer AL was added, thoroughly vortexed and incubated for 10 minutes at 70°C in a hot water bath. After 10 minutes of incubation, another 600 µl of one hundred percent ethanol (chilled) was added to precipitate the DNA bringing the total mixture to 1825 µl. The lysate was then added, 600µL at a time (3 times in total, each followed by spinning at 13,500 RPM for 1 minute) to a QIAamp spin column where the DNA is expected to settle. Thereafter, to wash and purify the DNA, wash buffers AW1 and AW2 (500 µl) were added in sequential order both centrifuged at 13,500 RPM for 1 minute and 3 minutes respectively. DNA was eluted to a total volume of 100 µl using AE buffer. All procedures followed were as per manufacturer’s instructions (Qiagen, 2014).

**Bone and hair samples:**

An initial of 0.5-1 cm cut from base of the hair (hair bulb) was taken into fresh 2ml tube. Similarly bone sample were wiped with ethanol and crushed into fine pieces and added into new 2 ml tubes to which 180 μl ATL (Tissue Lysis Buffer) and 20 μl Proteinase K Buffer (Protein degradation) was added and incubated at 56^o^C until the tissue was completely lysed. After 6-8 hours of complete tissue lysis, Buffer AL and chilled ethanol (200 μl each) were added and vortexed until thoroughly mixed. The sample was then collected in a DNeasy Mini Spin Column by centrifuging at 8,000 RPM for 1 minute with the lysate discarded. Two washing and purifying steps of washing buffers (AW1: low concentrate) and Buffer (AW2: High concentrate) at 500 μl each at 14,000 RPM was followed by elution using AE Buffer at 100 μl which was the final yield DNA extract.

**PCR amplification of D-loop segment:**

D-loop region (~530bp) in mtDNA was targeted using primers (YAK_Dloop_F2: 5’- GAGCCTCACCAGTATTAAATTT -3’ and YAK_Dloop_R2: 5’- ACAGTTATGTGTGAGCATGGGC -3’) designed at WildGenes, RZSS. PCRs were carried out in a 25 μl reaction with 12.5 μl Qiagen Mastermix, 2.5 μl Q-solution, 2 μl each Yak Dloop forward/reverse primers (10 μM) and 12 μl DNase free water. 3 μl of DNA template was added to each mix. Thermo-cycling conditions were initial denaturation of 95°C for 15 minutes followed by 40 cycles of 95°C for 30 seconds, annealing of 58°C for 30 seconds and elongation of 72°C for 60 seconds. Final elongation was 72°C for 10 minutes followed by a hold step at 16°C. The PCR products were electrophoresed on a 2% agarose gel, stained with Gel Red (0.5 mg/ml), and observed under a UV transilluminator.

**Table S1.** The D-loop sequences of 25 yak samples generated in this study and their secondary data. DY= Domestic Yak, WY= Wild Yak and UY=Unknown Yak.

| SN | GenBank accession | Location | Morphotype | Haplotype | Sample ID | Sample type |
| --- | --- | --- | --- | --- | --- | --- |
| 1 | MW048416 | Nepal (Humla) | WY | china21 | WF330 | Dung |
| 2 | MW048417 | Nepal (Humla) | WY | china21 | WF331 | Dung |
| 3 | MW048418 | Nepal (Dolpa) | DY | nep3 | WF479 | Hair |
| 4 | MW048419 | Nepal (Dolpa) | DY | nep5 | WF480 | Hair |
| 5 | MW048420 | Nepal (Dolpa) | DY | china43 | WF481 | Hair |
| 6 | MW048421 | Nepal (Dolpa) | DY | nep6 | WF482 | Hair |
| 7 | MW048422 | Nepal (Dolpa) | DY | china8 | WF484 | Hair |
| 8 | MW048423 | Nepal (Dolpa) | DY | nep1 | WF485 | Hair |
| 9 | MW048424 | Nepal (Mustang) | DY | china17 | YK01 | Dung |
| 10 | MW048425 | Nepal (Mustang) | DY | china7 | YK02 | Bone |
| 11 | MW048426 | Nepal (Mustang) | DY | nep2 | YK03 | Dung |
| 12 | MW048427 | Nepal (Mustang) | DY | china17 | YK04 | Dung |
| 13 | MW048428 | Nepal (Mustang) | DY | china43 | YK05 | Hair |
| 14 | MW048429 | Nepal (Mustang) | DY | china8 | YK06 | Hair |
| 15 | MW048430 | Nepal (Humla) | DY | china7 | YK08 | Dung |
| 16 | MW048431 | Nepal (Humla) | DY | china46 | YK09 | Hair |
| 17 | MW048432 | Nepal (Humla) | DY | china47 | YK10 | Hair |
| 18 | MW048433 | Nepal (Humla) | DY | china17 | YK11 | Bone |
| 19 | MW048434 | Nepal (Humla) | DY | china17 | YK13 | Dung |
| 20 | MW048435 | Nepal (Humla) | UY | nep7 | YK14 | Dung |
| 21 | MW048436 | Nepal (Humla) | WY | nep2 | YK15 | Hair |
| 22 | MW048437 | Nepal (Dolpa) | DY | nep5 | YK16 | Hair |
| 23 | MW048438 | Nepal (Dolpa) | DY | nep4 | YK17 | Hair |
| 24 | MW048439 | Nepal (Dolpa) | DY | china1 | YK18 | Hair |
| 25 | MW048440 | Nepal (Dolpa) | WY | china46 | YK19 | Bone |

**Table S2.** The reference sequences from China along with their haplotype names assigned during analysis in this study. DY=Domestic Yak and WY=Wild Yak

| S.N. | GenBank accession | Location | Morphotype | Haplotype |
| --- | --- | --- | --- | --- |
| 1 | GQ464116 | China | WY | china39 |
| 2 | GQ464117 | China | WY | china21 |
| 3 | GQ464118 | China | WY | china20 |
| 4 | GQ464119 | China | WY | china17 |
| 5 | GQ464120 | China | WY | china17 |
| 6 | GQ464121 | China | WY | china38 |
| 7 | GQ464122 | China | WY | china19 |
| 8 | GQ464123 | China | WY | china31 |
| 9 | GQ464124 | China | WY | china19 |
| 10 | GQ464125 | China | WY | china45 |
| 11 | GQ464126 | China | WY | china45 |
| 12 | GQ464127 | China | WY | china37 |
| 13 | GQ464128 | China | WY | china45 |
| 14 | GQ464129 | China | WY | china37 |
| 15 | GQ464130 | China | WY | china26 |
| 16 | GQ464131 | China | WY | china26 |
| 17 | GQ464132 | China | WY | china27 |
| 18 | GQ464133 | China | WY | china36 |
| 19 | GQ464134 | China | WY | china41 |
| 20 | GQ464135 | China | WY | china17 |
| 21 | GQ464136 | China | WY | china40 |
| 22 | GQ464137 | China | WY | china35 |
| 23 | GQ464138 | China | WY | china17 |
| 24 | GQ464139 | China | WY | china22 |
| 25 | GQ464140 | China | WY | china30 |
| 26 | GQ464141 | China | WY | china41 |
| 27 | GQ464142 | China | WY | china14 |
| 28 | GQ464143 | China | WY | china41 |
| 29 | GQ464144 | China | WY | china41 |
| 30 | GQ464145 | China | WY | china28 |
| 31 | GQ464146 | China | WY | china24 |
| 32 | GQ464147 | China | WY | china18 |
| 33 | GQ464148 | China | WY | china13 |
| 34 | GQ464149 | China | WY | china26 |
| 35 | GQ464150 | China | DY | china44 |
| 36 | GQ464151 | China | DY | china31 |
| 37 | GQ464152 | China | DY | china43 |
| 38 | GQ464153 | China | DY | china26 |
| 39 | GQ464154 | China | DY | china17 |
| 40 | GQ464155 | China | DY | china26 |
| 41 | GQ464156 | China | DY | china17 |
| 42 | GQ464157 | China | DY | china17 |
| 43 | GQ464158 | China | DY | china17 |
| 44 | GQ464159 | China | DY | china34 |
| 45 | GQ464160 | China | DY | china43 |
| 46 | GQ464161 | China | DY | china43 |
| 47 | GQ464162 | China | DY | china17 |
| 48 | GQ464163 | China | DY | china17 |
| 49 | GQ464164 | China | DY | china17 |
| 50 | GQ464165 | China | DY | china43 |
| 51 | GQ464166 | China | DY | china17 |
| 52 | GQ464167 | China | DY | china17 |
| 53 | GQ464168 | China | DY | china6 |
| 54 | GQ464169 | China | DY | china26 |
| 55 | GQ464170 | China | DY | china46 |
| 56 | GQ464171 | China | DY | china26 |
| 57 | GQ464172 | China | DY | china10 |
| 58 | GQ464173 | China | DY | china17 |
| 59 | GQ464174 | China | DY | china17 |
| 60 | GQ464175 | China | DY | china17 |
| 61 | GQ464176 | China | DY | china17 |
| 62 | GQ464177 | China | DY | china26 |
| 63 | GQ464178 | China | DY | china17 |
| 64 | GQ464179 | China | DY | china26 |
| 65 | GQ464180 | China | DY | china17 |
| 66 | GQ464181 | China | DY | china17 |
| 67 | GQ464182 | China | DY | china26 |
| 68 | GQ464183 | China | DY | china46 |
| 69 | GQ464184 | China | DY | china33 |
| 70 | GQ464185 | China | DY | china33 |
| 71 | GQ464186 | China | DY | china17 |
| 72 | GQ464187 | China | DY | china46 |
| 73 | GQ464188 | China | DY | china43 |
| 74 | GQ464189 | China | DY | china7 |
| 75 | GQ464190 | China | DY | china17 |
| 76 | GQ464191 | China | DY | china4 |
| 77 | GQ464192 | China | DY | china46 |
| 78 | GQ464193 | China | DY | china17 |
| 79 | GQ464194 | China | DY | china16 |
| 80 | GQ464195 | China | DY | china17 |
| 81 | GQ464196 | China | DY | china7 |
| 82 | GQ464197 | China | DY | china17 |
| 83 | GQ464198 | China | DY | china17 |
| 84 | GQ464199 | China | DY | china26 |
| 85 | GQ464200 | China | DY | china46 |
| 86 | GQ464201 | China | DY | china17 |
| 87 | GQ464202 | China | DY | china7 |
| 88 | GQ464203 | China | DY | china17 |
| 89 | GQ464204 | China | DY | china17 |
| 90 | GQ464205 | China | DY | china17 |
| 91 | GQ464206 | China | DY | china17 |
| 92 | GQ464207 | China | DY | china17 |
| 93 | GQ464208 | China | DY | china15 |
| 94 | GQ464209 | China | DY | china43 |
| 95 | GQ464210 | China | DY | china47 |
| 96 | GQ464211 | China | DY | china35 |
| 97 | GQ464212 | China | DY | china35 |
| 98 | GQ464213 | China | DY | china7 |
| 99 | GQ464214 | China | DY | china45 |
| 100 | GQ464215 | China | DY | china26 |
| 101 | GQ464216 | China | DY | china42 |
| 102 | GQ464217 | China | DY | china7 |
| 103 | GQ464218 | China | DY | china5 |
| 104 | GQ464219 | China | DY | china43 |
| 105 | GQ464220 | China | DY | china48 |
| 106 | GQ464221 | China | DY | china3 |
| 107 | GQ464222 | China | DY | china17 |
| 108 | GQ464223 | China | DY | china2 |
| 109 | GQ464224 | China | DY | china26 |
| 110 | GQ464225 | China | DY | china26 |
| 111 | GQ464226 | China | DY | china9 |
| 112 | GQ464227 | China | DY | china8 |
| 113 | GQ464228 | China | DY | china46 |
| 114 | GQ464229 | China | DY | china12 |
| 115 | GQ464230 | China | DY | china11 |
| 116 | GQ464231 | China | DY | china43 |
| 117 | GQ464232 | China | DY | china35 |
| 118 | GQ464233 | China | DY | china35 |
| 119 | GQ464234 | China | DY | china7 |
| 120 | GQ464235 | China | DY | china17 |
| 121 | GQ464236 | China | DY | china1 |
| 122 | GQ464237 | China | DY | china23 |
| 123 | GQ464238 | China | DY | china32 |
| 124 | GQ464239 | China | DY | china29 |
| 125 | GQ464240 | China | DY | china12 |
| 126 | GQ464241 | China | DY | china43 |
| 127 | GQ464242 | China | DY | china43 |
| 128 | GQ464243 | China | DY | china43 |
| 129 | GQ464244 | China | DY | china25 |
| 130 | GQ464245 | China | DY | china17 |
